# Left Atrial Structural and Functional Changes in Adults with Congenital Septal Defects and Paroxysmal Atrial Fibrillation

**DOI:** 10.3390/jcm13196023

**Published:** 2024-10-09

**Authors:** Anton V. Minaev, Marina Yu. Mironenko, Vera I. Dontsova, Yulia D. Pirushkina, Bektur Sh. Berdibekov, Alexander S. Voynov, Julia A. Sarkisyan, Elena Z. Golukhova

**Affiliations:** 1 A.N. Bakoulev National Medical Research Center of Cardiovascular Surgery, 121552 Moscow, Russia; 2 Outpatient Clinic #67 of the Moscow Department of Healthcare, 127083 Moscow, Russia; 3 Outpatient Clinic #195 of the Moscow Department of Healthcare, 127083 Moscow, Russia

**Keywords:** ACHD, atrial fibrillation, left atrial strain, atrial septal defect

## Abstract

**Aims**. To identify the difference between adult patients with septal defects and paroxysmal atrial fibrillation (AF) and patients without a history of arrhythmia using the left atrial (LA) volume and function parameters, to reveal the parameters associated with AF development. **Methods and results**. In this prospective study, 81 patients with septal defects and left-to-right shunts were enrolled between 2021 and 2023 and divided into two groups: with paroxysmal AF and without AF. Left atrial function was analyzed based on the indexed left atrial volumes (LAVI and preA-LAVI), ejection fraction (LAEF), expansion index (LAEI), reservoir (LAS-r), conduit (LAS-cd) and contractile (LAS-ct) strain, and stiffness index (LASI) using a Philips CVx3D ultrasound system (Philips, Amsterdam, The Netherlands) and corresponding software. In total, 26 patients with paroxysmal atrial fibrillation (mean age: 59.6 ± 11.7 years, female: 80.8%) and 55 patients with septal defects without any history of arrhythmias (mean age: 44.8 ± 11.6 years, female: 81.8%) were included. All patients were in the NYHA class I or II at baseline. Our findings demonstrated a significant difference between all LA function parameters in the two groups. Upon univariable analysis, the LAVI, preA-LAVI, LASI, LAEF, LAEI, LAS-r, LAS-c, LAS-ct, age, cardiac index, E/A, and RV pressure were found to be associated with AF. The multivariate analysis identified LAVI (OR 1.236, 95% CI 1.022–1.494, *p* = 0.03), LAS-r (OR 0.723, 95% CI 0.556–0.940, *p* = 0.02), and LAS-ct (OR 1.518, 95% CI 1.225–1.880, *p* < 0.001) as independent predictors of AF development. The proposed model demonstrated high sensitivity and specificity with an adjusted classification threshold of 0.38 (AUC: 0.97, 95% CI 0.93–1.00, sensitivity 92% and specificity 92%, *p* < 0.001). **Conclusions**. The assessment of LA function using speckle-tracking echocardiography demonstrated significantly different values in the AF group among patients with congenital septal defects. This technique can therefore be implemented in routine clinical management. **The key message.** Atrial fibrillation development in adult patients with congenital septal defects and a left-to-right shunt is associated with the changes in left atrial function under conditions of an increased preload.

## 1. Introduction

Untreated congenital heart defects (CHD) with intracardiac left-to-right shunts remain an actual problem of modern cardiology. Due to a long asymptomatic period, these patients seek medical attention in adulthood and even in old age. Long-term volume overload leads to atrial enlargement and fibrosis. Palpitation may be the manifesting symptom and is usually caused by atrial fibrillation. The incidence of atrial fibrillation in adult patients with congenital heart disease (ACHD) is significantly higher than in the general population and has a major impact on patient’s quality of life and prognosis [1,2].

The left atrium (LA) is a chamber that modulates left ventricular (LV) filling, and its cycle consists of reservoir, conduit, and contraction phases when in sinus rhythm. The remodeling of this chamber is one of the major factors leading to the development of atrial fibrillation. Left atrial dysfunction has been documented in patients with many cardiovascular diseases, such as hypertension, heart failure, valvular disease, etc. [3,4,5]. On the other hand, its function in patients with CHD remains unclear, especially in adults with arrhythmias. In this study, we used 2-dimensional speckle-tracking based strain imaging to evaluate the effect of LA deformation on LA volume and function parameters and to reveal the parameters associated with AF development.

## 2. Methods

### 2.1. Study Population

In total, 81 adult patients with septal defects (mean age 49.5 ± 13.4) were enrolled into the study at the Department of Congenital Heart Defects of A.N. Bakulev Center for Cardiovascular Surgery between 2021 and 2023. Congenital heart diseases were as follows: atrial septal defect (ASD, *n* = 70, 86.4%), partial anomalous pulmonary venous return with ASD (PAPVR, *n* = 7, 8.6%), ventricular septal defect (VSD, *n* = 1, 1.2%), partial atrioventricular septal defect (pAVSD, *n* = 3, 3.8%). These patients were divided into two groups: Group I comprised patients with at least one documented episode of atrial fibrillation who underwent spontaneous recovery or successful medical/electrical cardioversion; Group II (control) comprised patients without any history of arrhythmias. The first paroxysm of AF was documented in emergent ECG or Holter monitoring. There was no persistent arrhythmia or atrial flutter. The period from the first AF episode to LA assessment in Group I ranged between 2 and 8 months, with the number of short paroxysms from 1 to 4. All patients were considered for transcatheter or surgical treatment due to significant shunts and pulmonary overload. All patients were in a sinus rhythm at the time of the left atrial function evaluation. Prior to enrollment, all subjects provided written informed consent for participation in this study.

The exclusion criteria were as follows: previous ablation or cardiac surgery, mitral stenosis, ventricular hypoplasia, high pulmonary hypertension with pulmonary vascular resistance >5 WU, Qp/Qs < 1.5, inadequate 2D-STE myocardial tracking, HF with a reduced ejection fraction (EF), severe hepatic and renal dysfunction, and malignant tumor. 

### 2.2. Left Atrium Function Assessment

The Philips CVx3D ultrasound system (Philips, Amsterdam, The Netherlands) equipped with an X5-1 phased array transducer (1.0–5.0 MHz) and corresponding software package were utilized throughout the study. Gray-scale recordings were optimized at a mean frame rate of ≥50 frames/s. All echocardiographic measurements were performed in accordance with the guidelines of the American Society of Echocardiography [6], and the images and data were digitally stored for offline analysis. The experimental protocol was approved by a Bakulev Center licensing committee (#12–23). The left atrial function was evaluated using several parameters. The maximum volume index (LAVI) was measured prior to mitral valve opening at the ventricular end-systole and further indexed by the body surface area (BSA). The pre-A volume index (preA-LAVI) was measured prior to the atrial systole and further indexed based on the body surface area. The LA emptying fraction (LAEF) was calculated as the ratio between the volume of blood passed from the LA in one cycle and the maximal LA volume (in percents). The LA expansion index (LAEI) was calculated as the ratio between the volume of blood passed from the LA in one cycle and the minimal LA volume (in percents). The reservoir (LAS-r), conduit (LAS-c), and contractile (LAS-ct) strain were measured with two-dimensional speckle tracking echocardiography as the fractional change in the length of the entire atrial myocardium contour in the tangential direction. The stiffness index (LASI) was calculated as the ratio of early diastolic transmitral flow velocity/lateral mitral annulus myocardial velocity (E/e’) to LAS-r (that is, E/e’ divided by LAS-r). Three measurements for each patient were averaged and used for further analysis.

### 2.3. Statistics

The normality of the data distribution was assessed using the Kolmogorov–Smirnov test. Continuous variables with a normal distribution are presented as the means ± standard deviations, while categorical variables are reported as percentages. The SPSS software V28.0 was used for all statistical analyses. Clinical data, standard and speckle-tracking echocardiographic parameters, were compared between groups using Student’s unpaired *t*-test. Differences were considered statistically significant at *p* values < 0.05. To identify independent predictors of atrial fibrillation, univariate analysis and multivariate forward stepwise logistic regression analysis were used. Receiver operating characteristic curves were analyzed to determine the diagnostic accuracy for the prediction of atrial fibrillation development. 

## 3. Results

Eighty-one patients with septal defects were evaluated. Group I included 26 patients with septal defects with PAF; Group II included 55 patients with septal defects and no history of arrhythmia. Baseline demographic and clinical characteristics are presented in Table 1. The patients with PAF were significantly older than those without arrhythmia. The receiver operating characteristics (ROC) curve analysis revealed that the age of ≥54.5 years predicted a greater likelihood of AF development (area under the curve (AUC): 0.81, 95% CI 0.71–0.92, sensitivity 78% and specificity 76%, *p* < 0.001). There was no significant difference between the two groups in the body surface area, body mass index, heart rate, blood pressure, and pulmonary overload (Qp/Qs). However, diagnosed hypertension was more common in patients with AF. In addition, moderate mitral regurgitation and high right ventricular systolic pressure were observed in patients with PAF. Furthermore, the use of beta-blockers, amiodarone, and RAAS-blockers was more common in Group I. 

Continuous variables with a normal distribution are presented as the means ± standard deviations, and categorical variables are presented as percentages.

The left atrial function echocardiography parameters are presented in Table 2. A significant difference between all LA function parameters in the two groups was revealed. Consequently, LA volumes and the stiffness index were markedly elevated in patients with atrial fibrillation, whereas LAEF, LAEI, and strain (LAS-r, LAS-c, LAS-ct) were significantly diminished in this group. The ROC curve analysis revealed that LAVI ≥ 34.9 mL/m2 (AUC: 0.79, 95% CI 0.68–0.90, sensitivity 75% and specificity 72%, *p* < 0.001), preA-LAVI ≥ 23.8 mL/m2 (AUC: 0.80, 95% CI 0.70–0.90, sensitivity 75% and specificity 72%, *p* < 0.001), LAEF ≤ 59.2% (AUC: 0.78, 95% CI 0.76–0.94, sensitivity 72% and specificity 77%, *p* < 0.001), LAEI ≤ 145.7% (AUC: 0.78, 95% CI 0.66–0.89, sensitivity 72% and specificity 77%, *p* < 0.001), LAS-r ≤ 29.9% (AUC: 0.85, 95% CI 0.76–0.94, sensitivity 77% and specificity 80%, *p* < 0.001), LAS-ct ≤ 10.0% (AUC: 0.78, 95% CI 0.66–0.88, sensitivity 71% and specificity 76%, *p* < 0.001), and LASI ≥ 0.23 (AUC: 0.87, 95% CI 0.79–0.95, sensitivity 79% and specificity 80%, *p* < 0.001) allowed for the differentiation of the two groups and predicted patients more prone to AF.

Based on univariable analysis, LAVI, preA-LAVI, LASI, LAEF, LAEI, LAS-r, LAS-c, LAS-ct, age, CI, E/A, and RV pressure were associated with AF. 

The further multivariate logistic regression analysis discriminated only one factor associated with AF, LASI (*p* = 0.01, with an extremely high OR). After the removal of LASI from the previous model, LAVI (OR 1.236, 95% CI 1.022–1.494, *p* = 0.03), LAS-r (OR 0.723, 95% CI 0.556–0.940, *p* = 0.02), and LAS-ct (OR 1.518, 95% CI 1.225–1.880, *p* < 0.001) were identified as independent factors associated with AF development (Table 3). This model demonstrated high sensitivity and specificity with an adjusted classification threshold of 0.38 (AUC: 0.97, 95% CI 0.93–1.00, sensitivity 92% and specificity 92%, *p* < 0.001).

## 4. Discussion

In this study, we investigated the changes in left atrial (LA) function in patients with septal defects, pulmonary circulation overload, and paroxysmal atrial fibrillation compared with those without arrhythmia. The primary findings were as follows: all assessed parameters of LA function exhibited reliable differences between the two groups. High sensitivity and specificity were observed for LAVI, preA-LAVI, LAEF, LAEI, LAS-r, LAS-ct, and LASI. While LASI could be identified as the strongest predictor, in our study its significance was not confirmed due to statistical overload. The revealed independent factors associated with atrial fibrillation (AF) development with high sensitivity and specificity according to the proposed model were LAVI, LAS-r, and LAS-ct. It can be assumed that chronic volume overload leads to dilatation, progressive hypertrophy, and fibrosis of the left atrium resulting in AF development. In addition, some patients can be more susceptible to left atrial changes. These clinical characteristics can include age, hypertension, or high RV pressure, as was shown in this study.

The relationship between the left atrial volume and AF development is widely recognized. For example, a study has shown that an indexed LA volume increase of greater than 34.3 mL/m^2^ was a discriminatory node for AF development in patients with heart failure and preserved ejection fraction, in addition to LAS-c ≤ 12.7% and LAS-r ≤ 29.4% [7]. When discussing congenital septal defects, we refer to pulmonary circulation overload and an increase in pulmonary return to the left atrium. In adult patients with significant shunts, this leads to LA enlargement, which is uncommon in children. The increase in LA volume in adult patients with ASD has been demonstrated in several studies, with a subsequent reduction following ASD closure [8,9,10]. Conversely, increased LA in ACHD patients can be more prone to arrhythmia, which results in a higher incidence and younger age of AF in this population [1].

Nevertheless, LA volume was not different between patients with ASD who developed AF in 24 h after device closure and controls (26.4 ± 4.6 vs. 26.9 ± 4.9 mL/m^2^). In the aforementioned study (A. Vitarelli et al., 2018), the overall incidence of paroxysmal AF after 6 months of follow-up was 13% in PFO and 24% in ASD (8/58 vs. 14/58). Using the LA function parameter evaluation, the primary finding was that pre-closure 3D right and left atrial expansion indexes were independent predictors of AF based on multivariate analysis. The authors concluded that pre-closure atrial changes, more than device size, are implicated in paroxysmal AF development following atrial septal procedures [11].

In addition, another study showed no clear relation between closure of the ASD and AF development. In this cohort of 173 patients, ASD closure was performed. Almost 20% of patients developed AF. An older age and dilated left atrium were independently associated with AF, but ASD closure had no impact on the subsequent decrease in AF morbidity [12].

Another study evaluated LA function parameters to predict patients at a higher risk of developing postoperative AF after coronary artery bypass grafting. Patients with AF exhibited increased LA volumes, but multivariate regression analysis demonstrated that age and LAEF were strong independent predictors of postoperative AF with a cut-off point of LAEF ≤49.04%, determined via ROC analysis [13]. The findings of this study indicate that there is no universal parameter that can be used to predict AF development in all different evaluated groups. However, it can be concluded that LA function is significantly altered in patients who develop AF.

Our study corroborates the findings of previous research by demonstrating significant differences between cases with AF and controls in all presented parameters. The study included patients with septal defects and long-standing pulmonary overload, which determines increased left atrial preload. The predicting model and features in these patients remain unclear. It can be assumed that overload leads to LA enlargement and increased contractility, a process that is consistent with the Frank–Starling law. A study of the Frank–Starling law in the left atrium included 70 patients with varying LA preloads and volumes. The active atrial stroke volume was found to be significantly higher in the group with increased LA preload up to a point, beyond which LA contractility decreased [14]. In contrast, these changes were not substantiated in the right atrium [15]. In our study, all patients exhibited an increased LA preload, and we observed the difference between patients with AF and controls in terms of LAVI and LAEF. Consequently, LAVI in patients with AF was significantly higher regardless of a comparable pulmonary overload and Qp/Qs ratio. The LAEF was found to be lower in patients with AF, although in the group without arrhythmia, it can be higher than in the general population or patients with non-congenital diseases. For example, according to the literature, the mean LAEF in healthy controls varied from 50.6 to 65.8% [9,11,16], from 36.6 to 63.0% in patients with heart failure [5,7,17,18,19], and from 54.9 to 62.0% in patients with hypertension [3,20,21,22]. Our results demonstrated a mean range of 67.3% in patients without AF, which corroborates the previous conclusion about the applicability of the Frank–Starling law in LA with preserved function. 

## 5. Conclusions

The alterations in LA function parameters observed in ACHD patients with septal defects and pulmonary overload are most likely related to AF development and may serve as predictors of arrhythmia. In this population, speckle-tracking echocardiography may assist in assessing the risk of AF incidence. The clinical significance of atrial dysfunction should be further evaluated, particularly with regard to the definition of indications for antiarrhythmic treatment and ablation.

## 6. Limitations

The main limitation of this study was the relatively small sample size, in part determined by the availability of good-quality echocardiographic images. Other limitations include the small cohort, cofounders (such as mitral regurgitation), and statistical overload for LASI, which was removed from the multivariate analysis.

## Figures and Tables

**Table 1:** Demographic and clinical characteristics of patients with paroxysmal atrial fibrillation and controls. Continuous variables with a normal distribution are presented as the means ± standard deviations, and categorical variables are presented as percentages.

Variables	All (*n* = 81)	Group I (SD + PAF, *n* = 26)	Group II (SD without AF, *n* = 55)	*p*
Age (years)	49.5 ± 13.4	59.6 ± 11.7	44.8 ± 11.6	<0.001
Male (%)	18.5	19.2	18.2	0.91
Body mass index, (kg/m^2^)	26.6 ± 4.9	27.9 ± 5.0	26.0 ± 4.8	0.12
Body surface area, m^2^	1.8 ± 0.2	1.9 ± 0.2	1.8 ± 0.2	0.08
Heart rate, beats/m	73.7 ± 9.8	71.4 ± 6.9	74.7 ± 10.7	0.13
SBP, mm Hg	121.5 ± 11.5	122.9 ± 9.6	120.5 ± 12.7	0.48
Hypertension (%)	14.8	30.8	7.3	0.02
LVEDVI, mL/m^2^	51.5 ± 11.2	53.8 ± 9.1	49.6 ± 8.9	0.10
LVESVI, mL/m^2^	20.1 ± 3.4	21.2 ± 3.8	19.8 ± 4.0	0.27
LVEF, %	66.2 ± 5.6	66.3 ± 6.6	65.9 ± 5.0	0.81
LV cardiac index, l/min/m^2^	2.4 ± 0.6	2.7 ± 0.7	2.3 ± 0.7	0.02
Mitral E/A	1.2 ± 0.4	1.0 ± 0.3	1.2 ± 0.4	0.03
Mitral E/E′	7.0 ± 2.4	7.2 ± 2.0	6.8 ± 2.6	0.55
Moderate mitral regurgitation (%)	19.8	38.5	10.9	0.002
RV diastolic area, cm^2^	27.9 ± 5.0	29.6 ± 5.1	26.8 ± 4.2	0.08
Moderate or severe tricuspid regurgitation (%)	51.9	49.0	57.7	0.17
RV systolic pressure, mm Hg	42.8 ± 12.1	49.2 ± 14.2	38.9 ± 12.1	0.01
Qp/Qs	2.4 ± 0.7	2.5 ± 0.8	2.4 ± 0.7	0.79
Medications				
Diuretics (%)	41.9	53.8	36.4	0.14
β-Blockers (%)	13.6	30.8	5.5	0.01
Amiodarone (%)	19.7	61.5	0	<0.001
Calcium antagonists (%)	11.1	15.4	9.1	0.40
ACEIs or ARBs (%)	28.4	53.8	16.4	0.002

SD—septal defects, PAF—paroxysmal atrial fibrillation, SBP—systolic blood pressure, LVEDVI—left ventricular end-diastolic volume indexed, LVESVI—left ventricular end-systolic volume indexed, LVEF—left ventricular ejection fraction, RV—right ventricle.

**Table 2:** Speckle tracking deformation and volumetric parameters of the left atrial function in patients with paroxysmal atrial fibrillation and controls. The variables are presented as the mean ± standard deviation.

Variables	All (*n* = 81)	Group I (SD + PAF, *n* = 26)	Group II (SD without AF, *n* = 55)	*p*
LAVI (mL/m^2^)	36.7 ± 11.6	39.0 ± 13.3	28.3 ± 12.5	0.001
preA-LAVI (mL/m^2^)	22.3 ± 10.3	29.2 ± 10.2	18.9 ± 8.7	<0.001
LAEF (%)	62.9 ± 13.4	53.8 ± 13.2	67.3 ± 11.1	<0.001
LAEI (%)	211.6 ± 131.8	138.9 ± 85.6	246.5 ± 136.4	0.001
LAS-r (%)	31.9 ± 9.3	23.7 ± 7.6	35.2 ± 8.6	<0.001
LAS-c (%)	−20.5 ± 9.5	−15.6 ± 8.1	−22.9 ± 9.2	0.001
LAS-ct (%)	−11.7 ± 5.5	−8.2 ± 4.7	−13.4 ± 5.1	<0.001
LASI	0.21 ± 0.13	0.31 ± 0.15	0.17 ± 0.07	<0.001

LAVI—left atrial volume indexed; preA-LAVI—left atrial volume indexed before the atrial systole; LAEF—left atrial emptying fraction; LAEI—left atrial expansion index; LAS-r—left atrial reservoir strain; LAS-c—left atrial conduit strain; LAS-ct—left atrial contractile strain; LASI—left atrial stiffness index; SD—septal defects; PAF—paroxysmal atrial fibrillation.

**Table 3:** The results of logistic regression analysis.

Variables	Univariate		Multivariate	
	OR (95%CI)	*p*-Value	OR (95%CI)	*p*-Value
Age	1.116 (1.053–1.162)	*p* < 0.001	-	-
Male	0.933 (0.283–3.074)	*p* = 0.910	-	-
BMI	1.084 (0.979–1.200)	*p* = 0.119	-	-
LAVI	1.072 (1.025–1.121)	*p* = 0.002	1.236 (1.022–1.494)	*p* = 0.03
preA-LAVI	1.119 (1.051–1.191)	*p* < 0.001	-	-
LAEF	0.915 (0.873–0.959)	*p* < 0.001	-	-
LAEI	0.989 (0.983–0.996)	*p* = 0.001	-	-
LAS-r	0.856 (0.796–0.920)	*p* < 0.001	0.723 (0.556–0.940)	*p* = 0.02
LAS-c	0.910 (0.858–0.966)	*p* = 0.002	-	-
LAS-ct	1.235 (1.102–1.385)	*p* < 0.001	1.518 (1.225–1.880)	*p* < 0.001
CI	2.745 (1.080–6.979)	*p* = 0.03	-	-
E/A	0.204 (0.045–0.925)	*p* = 0.04	-	-
RV pressure	1.069 (1.008–1.133)	*p* = 0.03	-	-

BMI—body mass index, LAVI—left atrial volume indexed; preA-LAVI—left atrial volume indexed before the atrial systole; LAEF—left atrial emptying fraction; LAEI—left atrial expansion index; LAS-r—left atrial reservoir strain; LAS-c—left atrial conduit strain; LAS-ct—left atrial contractile strain; CI—cardiac index, RV—right ventricle.

## Data Availability

The datasets used and/or analyzed during the current study available from the corresponding author upon reasonable request. Registration of research studies. This study is not a ‘First in Man’ study. Written informed consent was obtained from the patient for publication of this case report and accompanying images. A copy of the written consent is available for review by the Editor-in-Chief of this journal on request. Provenance and peer review. Not commissioned, externally peer-reviewed.
